# Draft genome sequences of *Burkholderia* and *Paraburkholderia* strains isolated from *Panicum virgatum* soil and roots at the Lux Arbor Reserve in Michigan, USA

**DOI:** 10.1128/mra.00880-25

**Published:** 2025-10-10

**Authors:** Hanna Kehlet-Delgado, Renee H. Petipas, Amanda A. Antoch, Jeffrey S. Norman, Xiufen Li, Richard Allen White, Sarah E. Evans, Lisa K. Tiemann, Maren L. Friesen

**Affiliations:** 1 Department of Plant Pathology, Washington State University 6760, Pullman, Washington, USA; 2 Department of Plant Biology, University of Vermont 2092, Burlington, Vermont, USA; 3 Department of Microbiology, University of Washington 7284, Seattle, Washington, USA; 4 Biology Department, Swarthmore College 7761, Swarthmore, Pennsylvania, USA; 5 Department of Plant and Environmental Sciences, New Mexico State University 4423, Las Cruces, New Mexico, USA; 6 Department of Bioinformatics and Genomics, North Carolina Research Center (NCRC), The University of North Carolina at Charlotte, Kannapolis, North Carolina, USA; 7 Department of Bioinformatics and Genomics, Computational Intelligence to Predict Health and Environmental Risks (CIPHER), The University of North Carolina at Charlotte 14727, Charlotte, North Carolina, USA; 8 Department of Integrative Biology, Ecology, Evolution and Behavior Program, W.K. Kellogg Biological Station, Michigan State University 115976 , Hickory Corners, Michigan, USA; 9 Department of Plant, Soil, and Microbial Sciences, Michigan State University 3078, East Lansing, Michigan, USA; The University of Arizona, Tucson, Arizona, USA

**Keywords:** *Burkholderia*, *Paraburkholderia*, soil microbes, root microbes, switchgrass, *Panicum virgatum*

## Abstract

We present draft genomes of two *Paraburkholderia* strains and two *Burkholderia* strains. Three strains were isolated from surrounding soils and one from roots of switchgrass (*Panicum virgatum*) in Michigan, USA.

## ANNOUNCEMENT

Understanding the microbes associated with switchgrass, a dedicated biofuel crop, is critical to increase the sustainability of biofuel production. Root and soil samples used in isolations were collected from the switchgrass monoculture plots (treatment G5) at the Lux Arbor Reserve site (42.4764, −85.4519) of the Marginal Lands Experiment, established in 2015 as part of the Great Lakes Bioenergy Research Center in Michigan, USA. Here, there are four replicated blocks, each containing a switchgrass plot, and each plot contains sub-plots, treated with nitrogen at a rate of 56 kg/ha/year or no nitrogen. Soils at the site are Typic Hapludalfs (Alfisol) with a loam texture. Soils have a pH of 5.8, 0.77% total C, 0.06% total N, and 12 ppm inorganic phosphorus (1). *Paraburkholderia aspalathi* DN3004, *Paraburkholderia graminis* DN3005, and *Burkholderia* sp. DN3021 was isolated from 10-cm soil cores containing root material taken near switchgrass plants in unfertilized plots. *Burkholderia ambifaria* DN3045 was isolated from switchgrass roots collected from fertilized plots.

**Fig 1:**
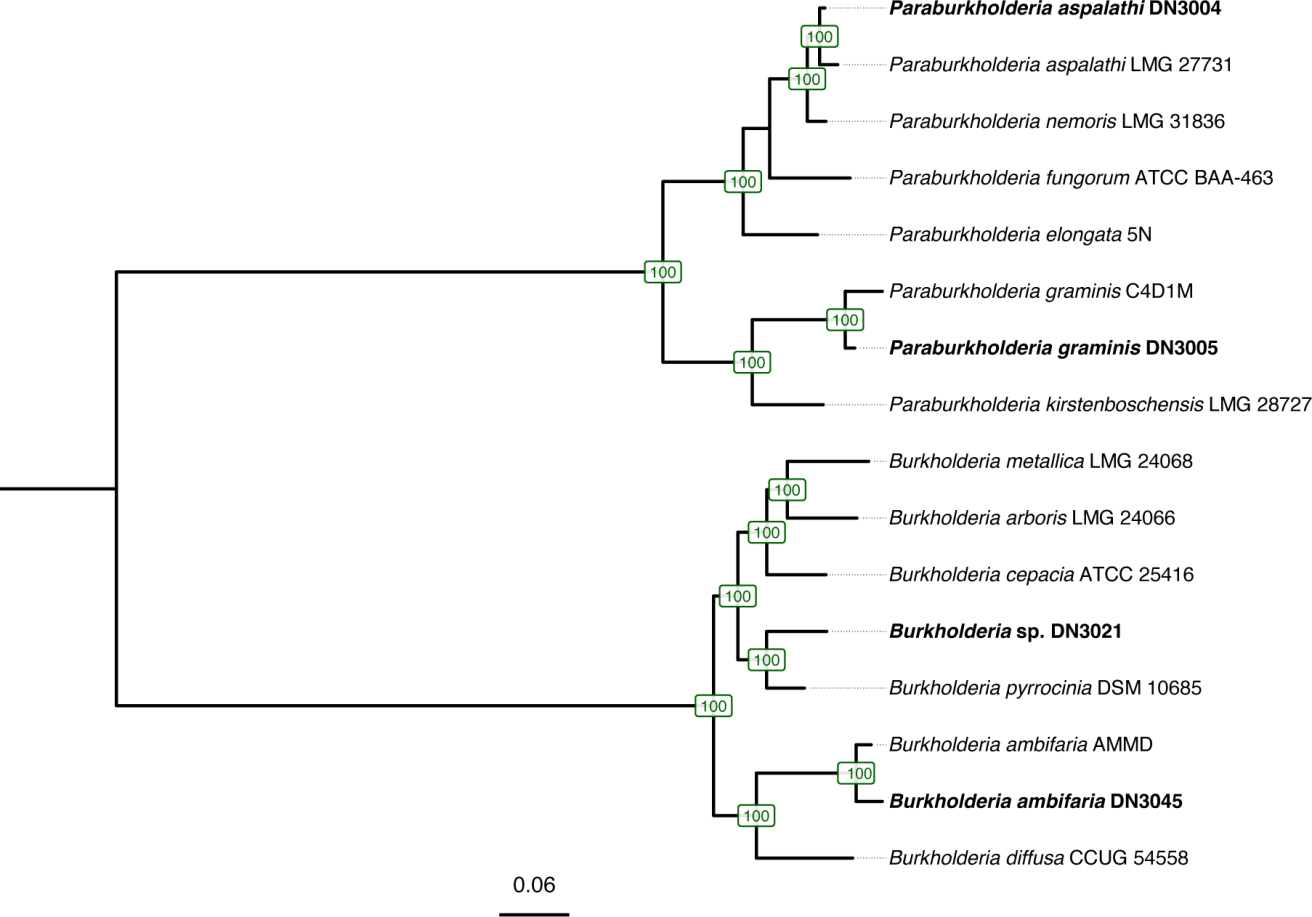
Phylogenetic relationships based on concatenated conserved marker gene sequences of *Burkholderia* and *Paraburkhoderia* strains isolated from switchgrass (indicated in bold) and related reference genomes were inferred through the maximum-likelihood method with RAxML v8.2.12 (15). Tree was re-rooted at the midpoint. Bootstrap values are displayed at nodes. Scale bar indicates amino acid substitutions per site.

Soil for isolations was suspended and diluted in 0.9% NaCl buffer. Strains DN3004 and DN3005 were isolated onto Burk’s Medium (2) made with sucrose (20 g/L). DN3021 was isolated onto Burk’s with sucrose in a 2% oxygen environment. For isolating DN3045, a one-inch segment of root was surface sterilized with bleach, rinsed with sterile water, and inserted into Burk’s soft agar with mannitol. Isolation protocols were adapted from reference (3). Isolates were cultured at 30°C. After recovery from frozen stocks (−80°C), plating on Burk’s agar with sucrose and 0.5 g/L NH_4_Cl, and incubation at 30°C, checking every 24 h until growth was evident, a single colony was selected to be grown under the same conditions for genomic DNA extraction from agar-grown colonies with the DNeasy PowerLyzer Microbial Kit (Qiagen, Germantown, MD, USA). DNA was purified using Sera-Mag Speedbeads carboxylated magnetic beads (Thermo Fisher Scientific, Waltham, MA, USA) and eluted in Tris buffer. Library preparation and whole genome sequencing were done at SeqCenter (Pittsburgh, PA). DNA libraries were prepared using the Illumina DNA Prep kit and IDT 10 bp UDI indices and sequenced on an Illumina NextSeq 2000, producing 2 × 151 bp reads. Demultiplexing, quality control, and adapter trimming were performed with bcl2fastq (v2.17; Illumina). Reads were assembled with Spades v3.14.1 (4) within Shovill (v. 1.1.0) (5) with the following options: genome size 7.5 Mb; minimum contig length 500 bp, “--trim,” and minimum coverage of 20×. Genome statistics was calculated with QUAST v5.2.0 (6). Genomes were assessed for completeness and contamination with CheckM v1.2.3 (7). Genomes were annotated using the NCBI Prokaryotic Genome Annotation Pipeline (PGAP) v6.10 (8). For taxonomic identification, we used the Type Strain Genome Server (TYGS) v391 to obtain digital DNA-DNA hybridization (dDDH) values with type strains (9, 10). Within PhyloPhlAn v3.0.67 (11), Diamond v2.1.8 (12) was used to perform a search against a database of 400 amino acid marker sequences universally conserved in bacteria (11, 13) using the options “--diversity medium” and “--accurate” against proteomes from genomes of the four newly sequenced strains and 12 reference *Burkholderia* and *Paraburkholderia* downloaded from NCBI. Marker genes were aligned using Mafft v7.520 (14), trimmed with trimAl “--gappyout” (15), and concatenated for phylogenetic reconstruction (Fig. 1) with RAxML v8.2.12 (16) under the model “PROTCATLG” with 1000 rapid bootstraps. Default parameters were used for all tools unless otherwise noted. Detailed genome information and accession numbers can be found in Table 1. These genomes may help expand our knowledge of potential growth-promoting bacteria associated with switchgrass.

**TABLE 1:** Genome assembly statistics for *Burkholderia* sp. DN3021, *B. ambifaria* DN3045, *P. aspalathi* DN3004, and *P. graminis* DN3005

Strain	DN3021	DN3045	DN3004	DN3005
Taxonomic designation	*Burkholderia sp*.	*Burkholderia ambifaria*	*Paraburkholderia aspalathi*	*Paraburkholderia graminis*
TYGS dDDH (d4 %) (type strain accession)	60.3 (GCA_001028665.1)	74.0 (GCA_000203915.1)	83.1 (GCA_900116445.1)	88.5 (GCA_000172415.1)
Num. reads	7,511,444	7,101,634	7,678,508	8,670,830
Size (bp)	7,830,195	7,165,103	8,487,362	7,184,575
Contigs (n)	292	133	53	39
GC content (%)	66.50	66.75	61.49	63.01
N50 (bp)	69,387	95,302	466,079	511,957
L50 (n)	34	23	7	5
Median (bp)	8,988	37,443	37,328	89,241
Completeness (%)	99.94	99.48	99.94	99.5
Contamination (%)	0.64	0.08	0.85	0
Coverage (x)	151	143	175	155
Num. coding sequences (total)	7,358	6,492	7,544	6,386
Num. RNAs (5S, 16S, 23S)	2, 2, 2	1, 2, 4	1, 1, 4	1, 1, 2
Num. tRNAs	61	58	55	52
Genbank accession	JBNCAB000000000	JBNCAA000000000	JBNCAD000000000	JBNCAC000000000
Assembly accession	GCA_049805555.1	GCA_049805535.1	GCA_049805615.1	GCA_049805575.1
SRA accession	SRX28421348	SRX28421349	SRX28421346	SRX28421347
BioSample accession	SAMN47444454	SAMN47444455	SAMN47444452	SAMN47444453

## Data Availability

The genomic data were submitted in GenBank under BioProject accession number PRJNA1237900. Individual Genome Accession, BioSample Accession, and Sequence Read Accession numbers can be found in Table 1. The versions described in this paper are the first versions.
